# Hypertension, Anxiety and Obstructive Sleep Apnea in Cardiovascular Disease and COVID-19: Mediation by Dietary Salt

**DOI:** 10.3390/diseases10040089

**Published:** 2022-10-16

**Authors:** Ronald B. Brown

**Affiliations:** School of Public Health Sciences, University of Waterloo, Waterloo, ON N2L 3G1, Canada; r26brown@uwaterloo.ca

**Keywords:** hypertension, anxiety, obstructive sleep apnea, cardiovascular disease, COVID-19, dietary salt, renin-angiotensin-aldosterone system, sympathetic nervous system, catecholamines, angiotensin II

## Abstract

This perspective paper used a grounded theory method to synthesize evidence proposing that sodium toxicity from excessive dietary salt intake is a potential common pathophysiological mechanism that mediates the association of hypertension, obstructive sleep apnea, and anxiety with cardiovascular disease and COVID-19. Increased anxiety in these conditions may be linked to a high-salt diet through stimulation of the sympathetic nervous system, which increases blood pressure while releasing catecholamines, causing a “fight or flight” response. A rostral shift of fluid overload from the lower to the upper body occurs in obstructive sleep apnea associated with COVID-19 and cardiovascular disease, and may be related to sodium and fluid retention triggered by hypertonic dehydration. Chronic activation of the renin-angiotensin-aldosterone system responds to salt-induced dehydration by increasing reabsorption of sodium and fluid, potentially exacerbating fluid overload. Anxiety may also be related to angiotensin II that stimulates the sympathetic nervous system to release catecholamines. More research is needed to investigate these proposed interrelated mechanisms mediated by dietary salt. Furthermore, dietary interventions should use a whole-food plant-based diet that eliminates foods processed with salt to test the effect of very low sodium intake levels on hypertension, anxiety, and obstructive sleep apnea in cardiovascular disease and COVID-19.

## 1. Introduction

Hypertension, or high blood pressure, is a leading risk factor for cardiovascular disease (CVD) [1], and hypertension’s impact on mortality in aging adults is predicted to increase over the next few decades [2]. Arterial pressure is regulated by lowering fluid retention and hypervolemia from high dietary salt intake [3]. The World Health Organization recommendations for salt reduction advise that “less than 5 g per day for adults helps to reduce blood pressure and risk of cardiovascular disease, stroke and coronary heart attack”; yet, “most people consume too much salt—on average 9–12 g per day, or around twice the recommended maximum level of intake” [4]. Other blood pressure mechanisms affected by excessive salt intake include vascular endothelial dysfunction [5], “changes in the structure and function of large elastic arteries”, as well as “modification in sympathetic activity, and in the autonomic neuronal modulation of the cardiovascular system” [6]. Blood pressure is reduced considerably in hypertensive patients when dietary salt intake is reduced, and smaller reductions occur in people with normal blood pressure [7]. Some individuals have higher sensitivity to the effects of dietary salt than other people, but “there is no consensus for a definition of salt sensitivity and the precise mechanisms that explain their association are not yet fully understood” [8]. Although prospective cohort studies have reported an increased association between prevalence of cardiovascular disease and low dietary salt intake, a review of these observational studies “indicates that the association is not causal” and is related to “invalid measurement of sodium intake and other methodologic limitations” [9].

Additional CVD risk factors include anxiety both as a comorbidity during prevalence of coronary heart disease (CHD) [10], and as a risk factor before incidence of CVD [11], although findings of anxiety independent of depression are inconsistent, and further research is needed [12]. Among subtypes of anxiety disorders, generalized anxiety disorder is most likely to increase the risk for major adverse cardiac events [13]. Characteristics of generalized anxiety disorder include “persistent, excessive, and unrealistic worry about everyday things” which is often uncontrollable and is “accompanied by many non-specific psychological and physical symptoms” [14]. Epidemiological surveys show that anxiety disorders affect up to 33.7% of the population at some point during their lifetime [15], and CVDs were responsible for 32% of global deaths in 2019 [16]. “Considering the massive impact of both anxiety disorders and CVD in terms of mortality and quality of life, further enquiry into a possible association between them appears both relevant and necessary [17]”.

Furthermore, a scientific statement from the American Heart Association notes that obstructive sleep apnea (OSA), “episodic cycles of breathing disruption”, increases risk of “all-cause mortality and cardiovascular mortality”, and is “often underrecognized and undertreated in cardiovascular practice” [18]. Collapse of the upper airway with decreased oxygen saturation occurs in OSA, however, “pharyngeal narrowing and closure during sleep is a complex phenomenon, and likely multiple factors play a role in the pathogenesis” [19]. OSA prevalence in the adult population ranges from 9% to 38%, and prevalence increases with “advancing age, male sex, and higher body-mass index” [20].

Coincidently, at the time of this publication, over a dozen studies have associated OSA with risk of COVID-19 [21,22,23,24,25,26,27,28,29,30,31,32,33,34], and many studies have associated COVID-19 with hypertension [35,36,37,38,39,40,41,42,43,44,45,46,47,48] and with preexisting and comorbid anxiety [49,50,51,52,53,54,55], although some findings of psychiatric disorders in COVID-19 are inconsistent [56]. Nevertheless, these relationships suggest that a common pathophysiological mechanism may mediate hypertension, anxiety, and OSA with increased risk for CVD and COVID-19. 

Of relevance, sodium toxicity, the toxic effects from acute sodium chloride poisoning [57] or more commonly from chronic dietary salt overload [58], is associated with the nutritional epidemiology and nutritional immunology of COVID-19 [59]. Summarizing briefly, hyponatremia is associated with COVID-19, but this may be due to hypervolemia from excessive salt and fluid intake. Pulmonary edema related to salt intake causes severe acute respiratory symptoms associated with SARS-CoV-2 infection, and sodium toxicity is also related to fever, nasal congestion, delayed viral clearance, a cytokine storm, and other immune responses in COVID-19 [59]. The present perspective article proposes a grounded theory that sodium toxicity from excessive dietary salt mediates the association of hypertension, anxiety, and obstructive sleep apnea as risk factors for CVD and COVID-19. 

## 2. Method

This perspective paper used a grounded theory method to rigorously review the research literature [60]. Starting with a clean slate to remove assumptions and increase objectively, relevant keyword searches of databases, including PubMed, Scopus, and Google Scholar, were used to retrieve information on a high salt diet, sodium toxicity, hypertension, anxiety, obstruction sleep apnea, cardiovascular disease, and COVID-19. Unlike a systematic review, a literature review in grounded theory may change selection criteria as the trail of evidence changes, a process known as theoretical sampling A comparative analysis of information was used to form concepts, and concepts were synthesized into themes and interrelationships in an iterative manner until an explanatory theory emerged. The grounded theory presented in this paper offers novel insights, new directions for further research, and a basis for future hypothesis testing.

## 3. Anxiety, CVD, and Sodium Toxicity

Diagnosis of anxiety according to criteria of the International Classification of Diseases-8 (ICD-8) was associated with an increased risk of subsequent coronary heart disease events in 49,321 Swedish men [61]. A 2010 meta-analysis of 20 studies found a higher incidence of coronary heart disease and cardiac mortality associated with anxiety, prompting the researchers to suggest that anxiety was an independent risk factor for cardiac morbidity and mortality [62]. More recently, a 2016 meta-analysis of 37 studies found that anxiety was associated with a 52% increased risk of CVD prevalence [63], and new onset CVD was associated with anxiousness in a 2020 study of a German population [64]. Nevertheless, the underlying mechanisms causatively linking anxiety with CVD are unknown. In an editorial in the Journal of the American College of Cardiology, Dimsdale [65] speculated that leading pathophysiological mediators in the causative pathway between anxiety and heart disease include “sympathetic nervous system activity and various inflammatory markers”. Dimsdale further noted the need to scrutinize potential underlying risk factors that are common to anxiety and CVD.

The adrenal catecholamines epinephrine and norepinephrine of the sympathetic nervous system increase the “fight-or-flight” stress response, and dysregulation of this response under conditions of chronic stress can contribute to anxiety [66]. A high-salt diet in a model of salt-sensitive mice was found to stimulate an overactive response of the sympathetic nervous system, which was associated with increased blood pressure and increased levels of adrenal epinephrine production [67]. Additionally, a systematic review and meta-analysis of epidemiological studies found that hypertension is associated with anxiety [68]. Hypertonic saline infusion also increased activity of the sympathetic nervous system and raised plasma norepinephrine levels in normal men [69]. Similarly, hypertonic saline induced panic attacks in an animal model of panic disorder [70]. This evidence suggests an anxiogenic link with high dietary salt intake, which hypothetically may satisfy criteria for toxin exposure in substance/medication-induced anxiety disorder, listed in the Diagnostic and Statistics Manuel-5 (DSM-5) [71]. Furthermore, an inflammatory response induced by high salt intake in healthy humans increases interleukin-6 (IL-6) and IL-23 pro-inflammatory cytokines, while reducing anti-inflammatory cytokine IL-10 [72].

## 4. OSA, Hypertension, and the Renin-Angiotensin-Aldosterone System

A nocturnal rostral shift that redistributes fluid overload from the lower body towards the head occurs in OSA [73], exacerbating obstruction in the upper airways and increasing blood pressure in patients with hypertension [74]. Secondary hyperaldosteronism, often present in OSA, occurs from excessive activation of the renin-angiotensin-aldosterone system (RAAS), which can be due to edematous disorders [75]. RAAS activation increases salt and fluid reabsorption in the kidneys which “is important for restoring homeostasis after dehydration”, and thirst responses to intracellular dehydration are mediated by angiotensin II type 2 receptors (AT_2_R) [76]. Importantly, infusion of hypertonic sodium chloride (hypernatremia) causes intracellular hypertonic dehydration [77], suggesting that excessive ingestion of sodium chloride and hypertonic dehydration could trigger RAAS activation and possibly chronic overcompensation as reabsorbed salt and fluid levels contribute to hypervolemia. Chronic RAAS activation causing tissue remodeling and dysfunction occurs in congestive heart failure, systemic hypertension, and chronic kidney disease [78]. Dysregulated RAAS response is also implicated in COVID-19 complications in patients with CVD [79]. The RAAS response related to edema and hypervolemia from high dietary salt intake could also explain excessive aldosterone levels associated with parapharyngeal edema and upper airway resistance in severe OSA [80]. 

Excessive salt consumed by 20 student volunteers in a study of OSA found that “the normal pattern of sleep was disturbed and the depth of sleep was decreased” [81]. A 2013 study found that OSA was prevalent in 77.3% of patients with resistant hypertension and hyperaldosteronism, and an increase in OSA severity was associated with high dietary salt intake [82]. The researchers hypothesized that high dietary salt intake was a causative factor in the study findings, and suggested that “interventional studies that use dietary salt restriction as a treatment strategy for OSA in patients with resistant hypertension and hyperaldosteronism are needed to test this hypothesis”. 

Subsequently, results of a randomized trial published in 2018 found only minor reductions in OSA severity after one week of testing the use of diuretics and reduced dietary sodium [83]. However, the sodium-restricted group in the study ingested a daily maximum intake of 3 g sodium, which is higher than the U.S. Dietary Reference Intake (DRI) of 2300 mg sodium advised to reduce chronic disease risk in adults, twice as high as the DRI of 1500 mg sodium considered adequate for adults [84], and six times higher than essential sodium requirements of 500 mg recommended by the U.S. National Heart, Lung, and Blood Institute [85]. Furthermore, a case–control study of sleep apnea in heart failure patients found that patients with sleep apnea had a mean daily sodium intake of 3000 mg compared to patients without sleep apnea with a mean daily sodium intake of 1900 mg [86]. More research is needed to test interventions with lower daily levels of sodium intake (500 mg–1500 mg) for OSA prevention.

## 5. OSA, Anxiety, and Angiotensin II

Anxiety is associated with OSA and sleep disorders [87,88,89,90,91,92,93,94,95,96], although causative relationships are not clear and require more investigations. Additionally, sympathetic nervous system response is increased and parasympathetic response is decreased in OSA, the opposite effect of normal sleep, and increased variability of heart rate and blood pressure often extends into daytime wakefulness with normal breathing [97], suggesting causative factors involving the RAAS response. 

**Figure 1:**
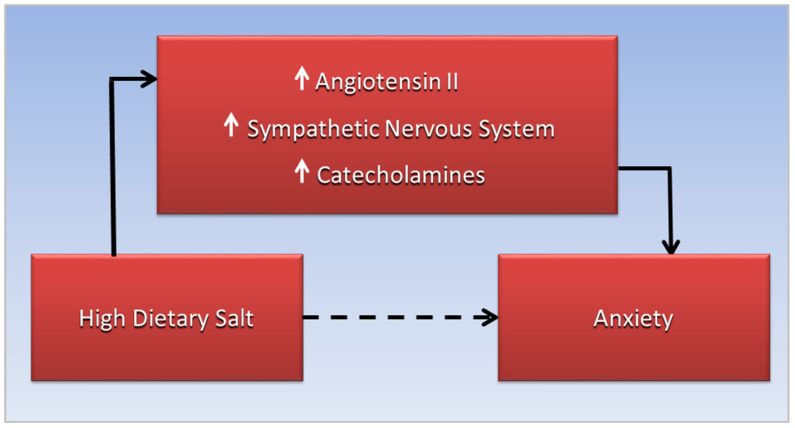
The association of high dietary salt and anxiety, the dotted line, is mediated by increased interaction of angiotensin II with the sympathetic nervous system, leading to increased catecholamine release.

Angiotensin II (AngII) of the RAAS response, derived from angiotensin I through action of angiotensin-I converting enzyme, increases blood pressure and retention of sodium and fluids, but as humans adopted a salt diet, the protective effects of the RAAS response turned into “a negative factor” [98]. “Plasma angiotensin II is increased in humans and animals with chronic heart failure” [99]. Additionally, AngII is “known to facilitate catecholamine release from peripheral sympathetic neurons by enhancing depolarization-dependent exocytosis”, contributing to vasoconstriction and sodium retention [100]. Of relevance, elevated levels of catecholamines are present in the urine and serum of patients with OSA, including children with OSA [101,102]. This evidence suggests that increased interaction of AngII with the sympathetic nervous system and increased release of catecholamines forms a potential mechanism that mediates the association of high dietary salt with anxiety, proposed in Figure 1. More research is needed to explore this anxiogenic mechanism.

## 6. Future Directions

Unlike clinical trials that have tested moderate reductions in sodium intake, future randomized trials are needed to test the effect of very low dietary sodium intake levels on CVD, COVID-19, OSA, and anxiety. For example, a recent randomized trial of patients with heart failure found no reduction in clinical events from a dietary intervention that moderately reduced sodium intake compared to a control group receiving usual care [103]. The mean sodium intake in the reduced-sodium group over 12 months was 1658 mg/day. Furthermore, the usual care group also reduced their sodium intake to 2073 mg/day, which is significantly lower than the average sodium intake of 3400 mg/day within the general U.S. population [104]. Future epidemiological studies should investigate much lower sodium intake levels, approximating the essential sodium dietary requirement of 500 mg, and test the effect of very low sodium intake levels against average levels within the general population. Of relevance, most dietary sodium intake comes from salt added to processed foods and from food consumed in restaurants [105]. To provide a very low level of dietary sodium, studies should eliminate all processed foods with added salt by employing whole-food plant-based diets, which have been found effective in reducing risks of COVID-19 [106].

To help consumers reduce dietary salt intake, the U.S. Centers for Disease Control and Prevention (CDC) recommends using salt-free seasonings when cooking, asking for food items prepared without salt when dining out, and carefully checking labels for low sodium when grocery shopping [107]. The CDC also recommends the Dietary Approaches to Stop Hypertension (DASH) eating plan which lowers dietary intake of salt and LDL-cholesterol [108]. Furthermore, “the most effective salt-reduction interventions have been implemented at the population level and comprise multi-component approaches, involving government, education and the food industry” [109].

**Figure 2:**
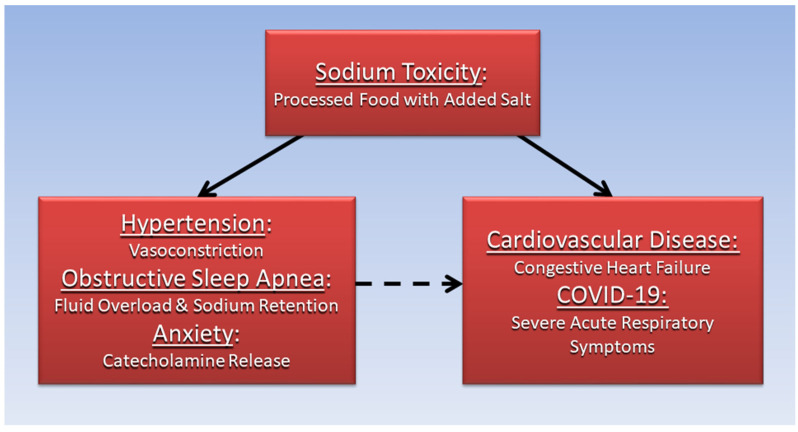
High salt diet and sodium toxicity mediates the association of hypertension, anxiety, and obstructive sleep apnea with cardiovascular disease and COVID-19.

Figure 2 summarizes this paper’s proposal that the association of CVD and COVID-19 with hypertension, OSA, and anxiety is mediated by high dietary salt and sodium toxicity. 

## 7. Conclusions

To summarize the proposal in this perspective paper: hypertension, anxiety, and obstructive sleep apnea are associated with cardiovascular disease and COVID-19. These associations are potentially mediated by high dietary salt intake and sodium toxicity, which stimulates the sympathetic nervous system to increase vascular restriction in hypertension, and retain fluid and sodium in obstructive sleep apnea. Hypertonic dehydration induced by high salt intake triggers the renin-angiotensin-aldosterone system to retain sodium and fluids, which may exacerbate fluid overload. Angiotensin II of the RAAS response also stimulates the sympathetic nervous system to release catecholamines which contributes to anxiety. Future studies should use a whole food plant-based diet to investigate the effect of very low dietary sodium levels on hypertension, anxiety, and obstructive sleep apnea in cardiovascular disease and COVID-19.

## References

[1] Fuchs F.D., Whelton P.K. (2020). High Blood Pressure and Cardiovascular Disease. Hypertension.

[2] Wu C.Y., Hu H.Y., Chou Y.J., Huang N., Chou Y.C., Li C.P. (2015). High Blood Pressure and All-Cause and Cardiovascular Disease Mortalities in Community-Dwelling Older Adults. Medicine.

[3] Hall J.E., Guyton A.C., Coleman T.G., Mizelle H.L., Woods L.L. (1986). Regulation of arterial pressure: Role of pressure natriuresis and diuresis. Fed. Proc..

[4] Who.Int Salt Reduction. https://www.who.int/news-room/fact-sheets/detail/salt-reduction.

[5] Choi H.Y., Park H.C., Ha S.K. (2015). Salt Sensitivity and Hypertension: A Paradigm Shift from Kidney Malfunction to Vascular Endothelial Dysfunction. Electrolytes Blood Press..

[6] Grillo A., Salvi L., Coruzzi P., Salvi P., Parati G. (2019). Sodium intake and hypertension. Nutrients.

[7] Rust P., Ekmekcioglu C., Islam M.S. (2017). Impact of Salt Intake on the Pathogenesis and Treatment of Hypertension. Hypertension: From Basic Research to Clinical Practice.

[8] Luzardo L., Noboa O., Boggia J. (2015). Mechanisms of Salt-Sensitive Hypertension. Curr. Hypertens. Rev..

[9] Cogswell M.E., Mugavero K., Bowman B.A., Frieden T.R. (2016). Dietary Sodium and Cardiovascular Disease Risk—Measurement Matters. N. Engl. J. Med..

[10] Chen Y.Y., Xu P., Wang Y., Song T.J., Luo N., Zhao L.J. (2019). Prevalence of and risk factors for anxiety after coronary heart disease: Systematic review and meta-analysis. Medicine.

[11] Allgulander C. (2016). Anxiety as a risk factor in cardiovascular disease. Curr. Opin. Psychiatry.

[12] Karlsen H.R., Saksvik-Lehouillier I., Stone K.L., Schernhammer E., Yaffe K., Langvik E. (2021). Anxiety as a risk factor for cardiovascular disease independent of depression: A prospective examination of community-dwelling men (the MrOS study). Psychol. Health.

[13] De Hert M., Detraux J., Vancampfort D. (2018). The intriguing relationship between coronary heart disease and mental disorders. Dialogues Clin. Neurosci..

[14] Munir S., Takov V., Coletti V.A. Generalized Anxiety Disorder. StatPearls [Internet]. https://www.ncbi.nlm.nih.gov/books/NBK441870/.

[15] Bandelow B., Michaelis S. (2015). Epidemiology of anxiety disorders in the 21st century. Dialogues Clin. Neurosci..

[16] Who.Int Cardiovascular Diseases (CVDs). https://www.who.int/news-room/fact-sheets/detail/cardiovascular-diseases-(cvds).

[17] Karlsen H.R., Matejschek F., Saksvik-Lehouillier I., Langvik E. (2021). Anxiety as a risk factor for cardiovascular disease independent of depression: A narrative review of current status and conflicting findings. Health Psychol. Open.

[18] Yeghiazarians Y., Jneid H., Tietjens J.R., Redline S., Brown D.L., El-Sherif N., Mehra R., Bozkurt B., Ndumele C.E., Somers V.K. (2021). Obstructive Sleep Apnea and Cardiovascular Disease: A Scientific Statement From the American Heart Association. Circulation.

[19] Slowik J.M., Sankari A., Collen J.F. Obstructive Sleep Apnea. StatPearls [Internet]. https://www.ncbi.nlm.nih.gov/books/NBK459252/.

[20] Senaratna C.V., Perret J.L., Lodge C.J., Lowe A.J., Campbell B.E., Matheson M.C., Hamilton G.S., Dharmage S.C. (2017). Prevalence of obstructive sleep apnea in the general population: A systematic review. Sleep Med. Rev..

[21] Cardoso E., Herrmann M.J., Grize L., Hostettler K.E., Bassetti S., Siegemund M., Khanna N., Sava M., Sommer G., Tamm M. (2022). Is sleep disordered breathing a risk factor for COVID-19 or vice versa?. ERJ Open Res..

[22] Kang H.H., Kim J.H., Kang B.J., Lee T., Ra S.W., Seo K.W., Jegal Y., Ahn J.J. (2021). Undiagnosed Obstructive Sleep Apnea and Acute COVID-19 Infection—A Case Series. Chronobiol. Med..

[23] Iannella G., Vicini C., Lechien J.R., Ravaglia C., Poletti V., di Cesare S., Amicarelli E., Gardelli L., Grosso C., Patacca A. (2021). Association Between Severity of COVID-19 Respiratory Disease and Risk of Obstructive Sleep Apnea. Ear Nose Throat J..

[24] Pena Orbea C., Wang L., Shah V., Jehi L., Milinovich A., Foldvary-Schaefer N., Chung M.K., Mashaqi S., Aboussouan L., Seidel K. (2021). Association of Sleep-Related Hypoxia With Risk of COVID-19 Hospitalizations and Mortality in a Large Integrated Health System. JAMA Netw. Open.

[25] Acet Öztürk N.A., Aydın Güçlü Ö., Alkan S., Şengören Dikiş Ö., Sali M., Yılmaz D., Taşbaş E., Ertem Cengiz A., Bahçetepe D., Aydın A. (2022). High-risk obstructive sleep apnea is related to longer hospital stay in COVID-19 patients. Eurasian J. Pulmonol..

[26] Hwang D., Shi J., Chen A., Arguelles J., Becker K.A., Kim J.B., Woodrum R.R., Valentine K., Benjafield A. (2021). Impact of Obstructive Sleep Apnea and Positive Airway Pressure Therapy on COVID-19 Outcomes. Am. J. Respir. Crit. Care Med..

[27] Labarca G., Henríquez-Beltrán M., Lamperti L., Nova-Lamperti E., Sanhueza S., Cabrera C., Quiroga R., Antilef B., Ormazábal V., Zúñiga F. (2022). Impact of Obstructive Sleep Apnea (OSA) in COVID-19 Survivors, Symptoms Changes Between 4-Months and 1 Year After the COVID-19 Infection. Front. Med..

[28] Voncken S.F.J., Feron T.M.H., Laven S., Karaca U., Beerhorst K., Klarenbeek P., Straetmans J., de Vries G.J., Kolfoort-Otte A.A.B., de Kruif M.D. (2022). Impact of obstructive sleep apnea on clinical outcomes in patients hospitalized with COVID-19. Sleep Breath..

[29] Maas M.B., Kim M., Malkani R.G., Abbott S.M., Zee P.C. (2021). Obstructive Sleep Apnea and Risk of COVID-19 Infection, Hospitalization and Respiratory Failure. Sleep Breath..

[30] Rögnvaldsson K.G., Eyþórsson E.S., Emilsson Ö.I., Eysteinsdóttir B., Pálsson R., Gottfreðsson M., Guðmundsson G., Steingrímsson V. (2022). Obstructive sleep apnea is an independent risk factor for severe COVID-19: A population-based study. Sleep.

[31] Hu M., Han X., Ren J., Wang Y., Yang H. (2022). Significant association of obstructive sleep apnoea with increased risk for fatal COVID-19: A quantitative meta-analysis based on adjusted effect estimates. Sleep Med. Rev..

[32] Cade B.E., Dashti H.S., Hassan S.M., Redline S., Karlson E.W. (2020). Sleep Apnea and COVID-19 Mortality and Hospitalization. Am. J. Respir. Crit. Care Med..

[33] Strausz S., Kiiskinen T., Broberg M., Ruotsalainen S., Koskela J., Bachour A., Palotie A., Palotie T., Ripatti S., Ollila H.M. (2021). Sleep apnoea is a risk factor for severe COVID-19. BMJ Open Respir. Res..

[34] Schwarzl G., Hayden M., Limbach M., Schultz K. (2021). The prevalence of Obstructive Sleep Apnea (OSA) in patients recovering from COVID-19. ERJ Open Res..

[35] WHO (2021). Hypertension and COVID-19: Scientific brief, 17 June 2021.

[36] Muhamad S.-A., Ugusman A., Kumar J., Skiba D., Hamid A.A., Aminuddin A. (2021). COVID-19 and Hypertension: The What, the Why, and the How. Front. Physiol..

[37] Akpek M. (2022). Does COVID-19 Cause Hypertension?. Angiology.

[38] Ebinger J., Driver M., Joung S., Tran T., Barajas D., Wu M., Botting P., Navarrette J., Sun N., Cheng S. (2022). Hypertension and Excess Risk for Severe COVID-19 Illness Despite Booster Vaccination. Hypertension.

[39] Chen J., Liu Y., Qin J., Ruan C., Zeng X., Xu A., Yang R., Li J., Cai H., Zhang Z. (2022). Hypertension as an independent risk factor for severity and mortality in patients with COVID-19: A retrospective study. Postgrad. Med. J..

[40] Bepouka B., Situakibanza H., Sangare M., Mandina M., Mayasi N., Longokolo M., Odio O., Mangala D., Isekusu F., Kayembe J.M. (2022). Mortality associated with COVID-19 and hypertension in sub-Saharan Africa. A systematic review and meta-analysis. J. Clin. Hypertens..

[41] Ribeiro A.C., Uehara S. (2022). Systemic arterial hypertension as a risk factor for the severe form of covid-19: Scoping review. Rev. De Saúde Pública.

[42] Wang J., Zhang Y., Li K., Du K., Huang X., Zhou Z., Ma Y., Guo S., Hou Y., Li Q. (2022). Retrospective Study of Aging and Sex-Specific Risk Factors of COVID-19 with Hypertension in China. Cardiovasc. Ther..

[43] Swamy S., Koch C.A., Hannah-Shmouni F., Schiffrin E.L., Klubo-Gwiezdzinska J., Gubbi S. (2022). Hypertension and COVID-19: Updates from the era of vaccines and variants. J. Clin. Transl. Endocrinol..

[44] Savoia C., Volpe M., Kreutz R. (2021). Hypertension, a Moving Target in COVID-19. Circ. Res..

[45] Clark C.E., McDonagh S.T.J., McManus R.J., Martin U. (2021). COVID-19 and hypertension: Risks and management. A scientific statement on behalf of the British and Irish Hypertension Society. J. Hum. Hypertens..

[46] Tadic M., Saeed S., Grassi G., Taddei S., Mancia G., Cuspidi C. (2021). Hypertension and COVID-19: Ongoing Controversies. Front. Cardiovasc. Med..

[47] Du Y., Zhou N., Zha W., Lv Y. (2021). Hypertension is a clinically important risk factor for critical illness and mortality in COVID-19: A meta-analysis. Nutr. Metab. Cardiovasc. Dis..

[48] Xia F., Zhang M., Cui B., An W., Chen M., Yang P., Qin T., Zhou X., Liao Y., Xu X. (2021). COVID-19 patients with hypertension are at potential risk of worsened organ injury. Sci. Rep..

[49] Wang Y., Yang Y., Ren L., Shao Y., Tao W., Dai X.J. (2021). Preexisting Mental Disorders Increase the Risk of COVID-19 Infection and Associated Mortality. Front. Public Health.

[50] Zhang S., Zhong Y., Wang L., Yin X., Li Y., Liu Y., Dai Q., Tong A., Li D., Zhang L. (2022). Anxiety, home blood pressure monitoring, and cardiovascular events among older hypertension patients during the COVID-19 pandemic. Hypertens. Res..

[51] Mazza M.G., De Lorenzo R., Conte C., Poletti S., Vai B., Bollettini I., Melloni E.M.T., Furlan R., Ciceri F., Rovere-Querini P. (2020). Anxiety and depression in COVID-19 survivors: Role of inflammatory and clinical predictors. Brain Behav. Immun..

[52] Li T., Sun S., Liu B., Wang J., Zhang Y., Gong C., Duan J. (2021). Prevalence and Risk Factors for Anxiety and Depression in Patients With COVID-19 in Wuhan, China. Psychosom. Med..

[53] Qiao S., Zhang J., Chen S., Olatosi B., Hardeman S., Narasimhan M., Bruner L., Diedhiou A., Scott C., Mansaray A. (2022). How Different Pre-existing Mental Disorders and Their Co-occurrence Affects COVID-19 Clinical Outcomes? A Real-World Data Study in the Southern United States. Front. Public Health.

[54] Taquet M., Luciano S., Geddes J.R., Harrison P.J. (2021). Bidirectional associations between COVID-19 and psychiatric disorder: Retrospective cohort studies of 62 354 COVID-19 cases in the USA. Lancet Psychiatry.

[55] Teixeira A.L., Krause T.M., Ghosh L., Shahani L., Machado-Vieira R., Lane S.D., Boerwinkle E., Soares J.C. (2021). Analysis of COVID-19 Infection and Mortality Among Patients With Psychiatric Disorders, 2020. JAMA Netw. Open.

[56] Luykx J.J., Lin B.D. (2021). Are psychiatric disorders risk factors for COVID-19 susceptibility and severity? a two-sample, bidirectional, univariable, and multivariable Mendelian Randomization study. Transl. Psychiatry.

[57] Metheny N.A., Krieger M.M. (2020). Salt Toxicity: A Systematic Review and Case Reports. J. Emerg. Nurs..

[58] Agócs R., Sugár D., Szabó A.J. (2020). Is too much salt harmful? Yes. Pediatr. Nephrol..

[59] Brown R.B. (2021). Sodium Toxicity in the Nutritional Epidemiology and Nutritional Immunology of COVID-19. Medicina.

[60] Wolfswinkel J.F., Furtmueller E., Wilderom C.P.M. (2013). Using grounded theory as a method for rigorously reviewing literature. Eur. J. Inf. Syst..

[61] Janszky I., Ahnve S., Lundberg I., Hemmingsson T. (2010). Early-Onset Depression, Anxiety, and Risk of Subsequent Coronary Heart Disease: 37-Year Follow-Up of 49,321 Young Swedish Men. J. Am. Coll. Cardiol..

[62] Roest A.M., Martens E.J., de Jonge P., Denollet J. (2010). Anxiety and risk of incident coronary heart disease: A meta-analysis. J. Am. Coll. Cardiol..

[63] Batelaan N.M., Seldenrijk A., Bot M., van Balkom A.J., Penninx B.W. (2016). Anxiety and new onset of cardiovascular disease: Critical review and meta-analysis. Br. J. Psychiatry.

[64] Reiner I.C., Tibubos A.N., Werner A.M., Ernst M., Brähler E., Wiltink J., Michal M., Schulz A., Wild P.S., Münzel T. (2020). The association of chronic anxiousness with cardiovascular disease and mortality in the community: Results from the Gutenberg Health Study. Sci. Rep..

[65] Dimsdale J.E. (2010). What does heart disease have to do with anxiety?. J. Am. Coll. Cardiol..

[66] Goddard A.W., Ball S.G., Martinez J., Robinson M.J., Yang C.R., Russell J.M., Shekhar A. (2010). Current perspectives of the roles of the central norepinephrine system in anxiety and depression. Depress. Anxiety.

[67] Ralph A.F., Grenier C., Costello H.M., Stewart K., Ivy J.R., Dhaun N., Bailey M.A. (2021). Activation of the Sympathetic Nervous System Promotes Blood Pressure Salt-Sensitivity in C57BL6/J Mice. Hypertension.

[68] Pan Y., Cai W., Cheng Q., Dong W., An T., Yan J. (2015). Association between anxiety and hypertension: A systematic review and meta-analysis of epidemiological studies. Neuropsychiatr. Dis. Treat..

[69] Peskind E.R., Radant A., Dobie D.J., Hughes J., Wilkinson C.W., Sikkema C., Veith R.C., Dorsa D.M., Raskind M.A. (1993). Hypertonic saline infusion increases plasma norepinephrine concentrations in normal men. Psychoneuroendocrinology.

[70] Molosh A.I., Johnson P.L., Fitz S.D., Dimicco J.A., Herman J.P., Shekhar A. (2010). Changes in central sodium and not osmolarity or lactate induce panic-like responses in a model of panic disorder. Neuropsychopharmacology.

[71] Arnold E. Anxiety DSM-5 Diagnostic Criteria and Treatment Overview. https://pro.psycom.net/assessment-diagnosis-adherence/anxiety.

[72] Yi B., Titze J., Rykova M., Feuerecker M., Vassilieva G., Nichiporuk I., Schelling G., Morukov B., Choukèr A. (2015). Effects of dietary salt levels on monocytic cells and immune responses in healthy human subjects: A longitudinal study. Transl. Res..

[73] da Silva B.C., Kasai T., Coelho F.M., Zatz R., Elias R.M. (2017). Fluid Redistribution in Sleep Apnea: Therapeutic Implications in Edematous States. Front. Med..

[74] Bangash A., Wajid F., Poolacherla R., Mim F.K., Rutkofsky I.H. (2020). Obstructive Sleep Apnea and Hypertension: A Review of the Relationship and Pathogenic Association. Cureus.

[75] Dominguez A., Muppidi V., Gupta S. Hyperaldosteronism. https://www.ncbi.nlm.nih.gov/books/NBK499983/.

[76] Coble J.P., Grobe J.L., Johnson A.K., Sigmund C.D. (2015). Mechanisms of brain renin angiotensin system-induced drinking and blood pressure: Importance of the subfornical organ. Am. J. Physiol. Regul. Integr. Comp. Physiol..

[77] Tiarks G. Hypertonic dehydration: What Is It, Causes, Treatment, and More. https://www.osmosis.org/answers/hypertonic-dehydration.

[78] Ames M.K., Atkins C.E., Pitt B. (2019). The renin-angiotensin-aldosterone system and its suppression. J. Vet. Intern. Med..

[79] Augustine R., Abhilash S., Nayeem A., Salam S.A., Augustine P., Dan P., Maureira P., Mraiche F., Gentile C., Hansbro P.M. (2022). Increased complications of COVID-19 in people with cardiovascular disease: Role of the renin–angiotensin-aldosterone system (RAAS) dysregulation. Chem. Biol. Interact..

[80] Dudenbostel T., Calhoun D.A. (2012). Resistant hypertension, obstructive sleep apnoea and aldosterone. J. Hum. Hypertens..

[81] Fereidoun H., Pouria H. (2014). Effect of excessive salt consumption on night’s sleep. Pak J. physiol..

[82] Pimenta E., Stowasser M., Gordon R.D., Harding S.M., Batlouni M., Zhang B., Oparil S., Calhoun D.A. (2013). Increased dietary sodium is related to severity of obstructive sleep apnea in patients with resistant hypertension and hyperaldosteronism. Chest.

[83] Fiori C.Z., Martinez D., Montanari C.C., Lopez P., Camargo R., Sezerá L., Gonçalves S.C., Fuchs F.D. (2018). Diuretic or sodium-restricted diet for obstructive sleep apnea—A randomized trial. Sleep.

[84] Oria M., Harrison M., Stallings V.A., National Academies of Sciences, Engineering, and Medicine, Health and Medicine Division, Food and Nutrition Board, Committee to Review the Dietary Reference Intakes for Sodium and Potassium (2019). The National Academies Collection: Reports funded by National Institutes of Health. Dietary Reference Intakes for Sodium and Potassium.

[85] NHLBI (1996). Implementing Recommendations for Dietary Salt Reduction: Where Are We? Where Are We Going? How Do We Get There?: A Summary of an NHLBI Workshop.

[86] Kasai T., Arcand J., Allard J.P., Mak S., Azevedo E.R., Newton G.E., Bradley T.D. (2011). Relationship between sodium intake and sleep apnea in patients with heart failure. J. Am. Coll. Cardiol..

[87] Kim J.Y., Ko I., Kim D.K. (2019). Association of Obstructive Sleep Apnea With the Risk of Affective Disorders. JAMA Otolaryngol. –Head Neck Surg..

[88] Rezaeitalab F., Moharrari F., Saberi S., Asadpour H., Rezaeetalab F. (2014). The correlation of anxiety and depression with obstructive sleep apnea syndrome. J. Res. Med. Sci..

[89] Cox R.C., Olatunji B.O. (2020). Sleep in the anxiety-related disorders: A meta-analysis of subjective and objective research. Sleep Med. Rev..

[90] Akberzie W., Hesselbacher S., Aiyer I., Surani S., Surani Z.S. (2020). The Prevalence of Anxiety and Depression Symptoms in Obstructive Sleep Apnea. Cureus.

[91] Duan X., Zheng M., Zhao W., Huang J., Lao L., Li H., Lu J., Chen W., Liu X., Deng H. (2022). Associations of Depression, Anxiety, and Life Events With the Risk of Obstructive Sleep Apnea Evaluated by Berlin Questionnaire. Front. Med..

[92] Daabis R., Gharraf H. (2012). Predictors of anxiety and depression in patients with obstructive sleep apnea. Egypt. J. Chest Dis. Tuberc..

[93] Garbarino S., Bardwell W.A., Guglielmi O., Chiorri C., Bonanni E., Magnavita N. (2020). Association of Anxiety and Depression in Obstructive Sleep Apnea Patients: A Systematic Review and Meta-Analysis. Behav. Sleep Med..

[94] Batzikosta A., Antoniadou M., Tiga P., Nena E., Xanthoudaki M., Voulgaris A., Sotiropoulou R., Kouratzi M., Froudarakis M., Steiropoulos P. (2016). AB011. Assessment of anxiety and depressive symptoms in obstructive sleep apnea patients. Ann. Transl. Med..

[95] Wong J.L., Martinez F., Aguila A.P., Pal A., Aysola R.S., Henderson L.A., Macey P.M. (2021). Stress in obstructive sleep apnea. Sci. Rep..

[96] Sharafkhaneh A., Giray N., Richardson P., Young T., Hirshkowitz M. (2005). Association of psychiatric disorders and sleep apnea in a large cohort. Sleep.

[97] Mansukhani M.P., Kara T., Caples S.M., Somers V.K. (2014). Chemoreflexes, sleep apnea, and sympathetic dysregulation. Curr. Hypertens. Rep..

[98] Benigni A., Cassis P., Remuzzi G. (2010). Angiotensin II revisited: New roles in inflammation, immunology and aging. EMBO Mol. Med..

[99] Wang Y., Seto S.W., Golledge J. (2014). Angiotensin II, sympathetic nerve activity and chronic heart failure. Heart Fail. Rev..

[100] Dendorfer A., Thornagel A., Raasch W., Grisk O., Tempel K., Dominiak P. (2002). Angiotensin II induces catecholamine release by direct ganglionic excitation. Hypertension.

[101] Hakim F., Gozal D., Kheirandish-Gozal L. (2012). Sympathetic and catecholaminergic alterations in sleep apnea with particular emphasis on children. Front. Neurol..

[102] Sica E., De Bernardi F., Nosetti L., Martini S., Cosentino M., Castelnuovo P., Marino F. (2021). Catecholamines and children obstructive sleep apnea: A systematic review. Sleep Med..

[103] Ezekowitz J.A., Colin-Ramirez E., Ross H., Escobedo J., Macdonald P., Troughton R., Saldarriaga C., Alemayehu W., McAlister F.A., Arcand J. (2022). Reduction of dietary sodium to less than 100 mmol in heart failure (SODIUM-HF): An international, open-label, randomised, controlled trial. The Lancet.

[104] Patel Y., Joseph J. (2020). Sodium Intake and Heart Failure. Int. J. Mol. Sci..

[105] Harnack L.J., Cogswell M.E., Shikany J.M., Gardner C.D., Gillespie C., Loria C.M., Zhou X., Yuan K., Steffen L.M. (2017). Sources of Sodium in US Adults From 3 Geographic Regions. Circulation.

[106] Brown R.B. (2022). Low dietary sodium potentially mediates COVID-19 prevention associated with whole-food plant-based diets. Br. J. Nutr..

[107] cdc.gov How To Reduce Sodium Intake. https://www.cdc.gov/salt/reduce_sodium_tips.htm.

[108] nhlbi.nih.gov DASH Eating Plan. https://www.nhlbi.nih.gov/education/dash-eating-plan.

[109] Hunter R.W., Dhaun N., Bailey M.A. (2022). The impact of excessive salt intake on human health. Nat. Rev. Nephrol..

